# 5-(4-Chloro­phen­yl)-3-(2,4-dimethyl­thiazol-5-yl)-1,2,4-triazolo[3,4-*a*]isoquinoline

**DOI:** 10.1107/S160053681001278X

**Published:** 2010-04-10

**Authors:** F. Nawaz Khan, P. Manivel, K. Prabakaran, Venkatesha R. Hathwar, Mehmet Akkurt

**Affiliations:** a Organic and Medicinal Chemistry Research Laboratory, Organic Chemistry Division, School of Advanced Sciences, VIT University, Vellore 632 014, Tamil Nadu, India; b Solid State and Structural Chemistry Unit, Indian Institute of Science, Bangalore 560 012, Karnataka, India; c Department of Physics, Faculty of Arts and Sciences, Erciyes University, 38039 Kayseri, Turkey

## Abstract

In the title mol­ecule, C_21_H_15_ClN_4_S, the triazoloisoquinoline ring system is approximately planar, with an r.m.s. deviation of 0.054 (2) Å and a maximum deviation of 0.098 (2) Å from the mean plane for the triazole ring C atom that is bonded to the thia­zole ring. The thia­zole and benzene rings are twisted by 66.36 (7) and 56.32 (7)°, respectively, with respect to the mean plane of the triazoloisoquinoline ring system. In the crystal structure, mol­ecules are linked by inter­molecular C—H⋯N inter­actions along the *a* axis. The mol­ecular conformation is stabilized by a weak intra­molecular π–π inter­action involving the thia­zole and benzene rings, with a centroid–centroid distance of 3.6546 (11) Å. In addition, two other intermolecular π–π stacking inter­actions are observed, between the triazole and benzene rings and between the dihydro­pyridine and benzene rings [centroid–centroid distances = 3.6489 (11) and 3.5967 (10) Å, respectively].

## Related literature

For the synthesis and anti­helmintic activity of triazolo compounds similar to the title compound, see: Nadkarni *et al.* (2001 ▶). For related structures, see: Hui *et al.* (1999 ▶); Khan *et al.* (2010 ▶); Zou *et al.* (2004 ▶).
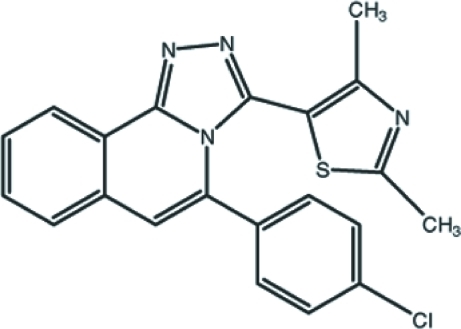


## Experimental

### 

#### Crystal data

C_21_H_15_ClN_4_S
*M* *_r_* = 390.89
Triclinic, 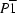

*a* = 7.8286 (5) Å
*b* = 8.1754 (6) Å
*c* = 15.1264 (9) Å
α = 93.514 (5)°
β = 94.805 (5)°
γ = 105.963 (6)°
*V* = 923.92 (11) Å^3^
*Z* = 2
Mo *K*α radiation
μ = 0.33 mm^−1^
*T* = 290 K
0.40 × 0.25 × 0.24 mm

#### Data collection

Oxford Xcalibur diffractometer with an Eos (Nova) CCD detector
Absorption correction: multi-scan (*CrysAlis PRO RED*; Oxford Diffraction, 2009 ▶) *T*_min_ = 0.851, *T*_max_ = 0.924
19579 measured reflections
3439 independent reflections
2518 reflections with *I* > 2σ(*I*)
*R*_int_ = 0.035

#### Refinement

*R*[*F* ^2^ > 2σ(*F* ^2^)] = 0.037
*wR*(*F* ^2^) = 0.111
*S* = 1.09
3439 reflections
246 parameters
H-atom parameters constrained
Δρ_max_ = 0.20 e Å^−3^
Δρ_min_ = −0.21 e Å^−3^

### 

Data collection: *CrysAlis PRO CCD* (Oxford Diffraction, 2009 ▶); cell refinement: *CrysAlis PRO CCD*; data reduction: *CrysAlis PRO RED* (Oxford Diffraction, 2009 ▶); program(s) used to solve structure: *SHELXS97* (Sheldrick, 2008 ▶); program(s) used to refine structure: *SHELXL97* (Sheldrick, 2008 ▶); molecular graphics: *ORTEP-3* (Farrugia, 1997 ▶); software used to prepare material for publication: *WinGX* (Farrugia, 1999 ▶).

## Supplementary Material

Crystal structure: contains datablocks global, I. DOI: 10.1107/S160053681001278X/fj2293sup1.cif

Structure factors: contains datablocks I. DOI: 10.1107/S160053681001278X/fj2293Isup2.hkl

Additional supplementary materials:  crystallographic information; 3D view; checkCIF report

## Figures and Tables

**Table 1:** Hydrogen-bond geometry (Å, °)

*D*—H⋯*A*	*D*—H	H⋯*A*	*D*⋯*A*	*D*—H⋯*A*
C6—H6⋯N2^i^	0.93	2.62	3.495 (2)	158
C8—H8⋯N3^i^	0.93	2.51	3.383 (2)	156

Symmetry code: (i) 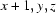.

**Fig. 1.**
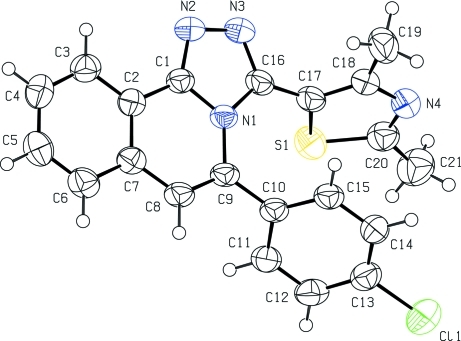
The title molecule with the atom numbering scheme. Displacement ellipsoids for non-H atoms are drawn at the 50% probability level.

**Fig. 2.**
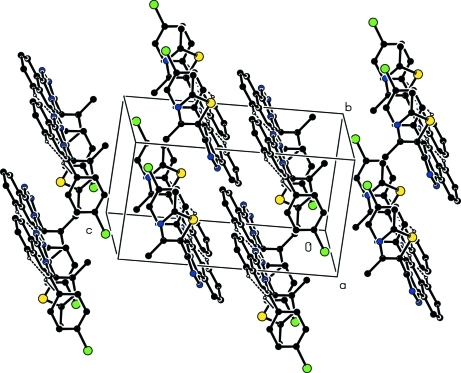
View of the packing diagram and the hydrogen bonding of (I) down the [100] direction. H atoms not involved in the motif shown have been omitted for clarity.

## supplementary crystallographic information

### Comment

Drugs including alprazolam (tranquilizer), estazolam (hypnotic, sedative, tranquilizer), rilmazafon (hypnotic, anxiolytic, used in the case of neurotic insomnia), benatradin (diuretic), trapidil (hypotensive), trazodon (antidepressant, anxiolytic), etoperidone (antidepressant), nefazodone (antidepressant, 5-HT2 A-antagonist), anastazole (antineoplastic, non-steroidal aromatase inhibitor), letrozole (antineoplastic, aromatase inhibitor), ribavirin (antiviral), fluconazole, itraconazole, terconazole (antifungal) possess 1,2,4-Triazole as the structural element. Besides, it follows from the literature data that 1,2,4-triazoles and their fused systems show antibacterial, antifungal and antiflammatory properties. As part of our search for new isoquinoline analogues, we focused on synthesis of titled compounds and the crystal structure is reported.

In the title molecule (I), Fig. 1, the triazoloisoquinoline ring system (N1–N3/C1–C9/C16) is nearly planar, with an r.m.s. deviation of 0.054 (2) Å and a maximum deviation of 0.098 (2) Å from the mean plane for the triazole ring C16 atom which is bonded to the thiazole ring (S1/N4/C17/C18/C20). The thiazole (S1/N4/C17/C18/C20) and benzene (C10—C15) rings are twisted by 66.36 (7) and 56.32 (7)°, respectively, with respect to the mean plane of the triazoloisoquinoline ring system. The thiazole ring forms a dihedral angle of 23.34 (9)° with benzene ring.

In the crystal structure of (I), molecules are linked by intermolecular C—H···N interactions along the [100] direction (Table 1, Fig. 2). Furthermore, π-π interactions [Cg1···Cg5(x, y, z) = 3.6546 (11) Å and Cg2···Cg4(2-x, 2-y, 1-z) = 3.6489 (11) Å. Where Cg1, Cg2, Cg4 and Cg5 are the centroids of the S1/N4/C17/C18/C20, N1–N3/C1/C16, C2–C7 and C10–C15 rings, respectively] are observed.

### Experimental

2-(3-(4-Chlorophenylisoquinolin-1-yl)hydrazine (1 mmol) was condensed with 2,4-dimethylthiazole-5-carbaldehyde (1.1 mmol) under refluxing conditions in isopropanol (10 ml) solvent to give the corresponding hydrazone in high yield. After removal of the solvent the compound was then oxidatively cyclized in nitrobenzene (10 ml) at 473 K. The product was recrystallized from dichlomethane to give block-shaped crystals.

### Refinement

All H atoms were placed in calculated positions with C–H = 0.93 and 0.96 Å and were included in the refinement in the riding model approximation, with *U_iso_*(H) = 1.2 or 1.5*U_eq_*(C).

### Crystal data

**Table:**

C_21_H_15_ClN_4_S	*Z* = 2
*M**_r_* = 390.89	*F*(000) = 404
Triclinic, *P*1	*D*_x_ = 1.405 Mg m^−^^3^
Hall symbol: -P 1	Mo *K*α radiation, λ = 0.71073 Å
*a* = 7.8286 (5) Å	Cell parameters from 954 reflections
*b* = 8.1754 (6) Å	θ = 2.0–20.4°
*c* = 15.1264 (9) Å	µ = 0.33 mm^−^^1^
α = 93.514 (5)°	*T* = 290 K
β = 94.805 (5)°	Block, pale yellow
γ = 105.963 (6)°	0.40 × 0.25 × 0.24 mm
*V* = 923.92 (11) Å^3^	

### Data collection

**Table:**

Oxford Xcalibur Eos (Nova) CCD detector diffractometer	3439 independent reflections
Radiation source: Enhance (Mo) X-ray Source	2518 reflections with *I* > 2σ(*I*)
graphite	*R*_int_ = 0.035
ω scans	θ_max_ = 25.5°, θ_min_ = 3.0°
Absorption correction: multi-scan (*CrysAlis PRO* RED; Oxford Diffraction, 2009)	*h* = −9→9
*T*_min_ = 0.851, *T*_max_ = 0.924	*k* = −9→9
19579 measured reflections	*l* = −18→18

### Refinement

**Table:**

Refinement on *F*^2^	Primary atom site location: structure-invariant direct methods
Least-squares matrix: full	Secondary atom site location: difference Fourier map
*R*[*F*^2^ > 2σ(*F*^2^)] = 0.037	Hydrogen site location: inferred from neighbouring sites
*wR*(*F*^2^) = 0.111	H-atom parameters constrained
*S* = 1.09	*w* = 1/[σ^2^(*F*_o_^2^) + (0.0635*P*)^2^] where *P* = (*F*_o_^2^ + 2*F*_c_^2^)/3
3439 reflections	(Δ/σ)_max_ < 0.001
246 parameters	Δρ_max_ = 0.20 e Å^−^^3^
0 restraints	Δρ_min_ = −0.20 e Å^−^^3^

### Special details

**Table:**

Geometry. All esds (except the esd in the dihedral angle between two l.s. planes) are estimated using the full covariance matrix. The cell esds are taken into account individually in the estimation of esds in distances, angles and torsion angles; correlations between esds in cell parameters are only used when they are defined by crystal symmetry. An approximate (isotropic) treatment of cell esds is used for estimating esds involving l.s. planes.
Refinement. Refinement of *F*^2^ against ALL reflections. The weighted *R*-factor wR and goodness of fit *S* are based on *F*^2^, conventional *R*-factors *R* are based on *F*, with *F* set to zero for negative *F*^2^. The threshold expression of *F*^2^ > σ(*F*^2^) is used only for calculating *R*-factors(gt) etc. and is not relevant to the choice of reflections for refinement. *R*-factors based on *F*^2^ are statistically about twice as large as those based on *F*, and *R*- factors based on ALL data will be even larger.

### Fractional atomic coordinates and isotropic or equivalent isotropic displacement parameters (Å^2^)

**Table:**

	*x*	*y*	*z*	*U*_iso_*/*U*_eq_	
S1	0.55766 (7)	0.44377 (7)	0.27355 (3)	0.05951 (19)	
Cl1	0.90834 (9)	0.16939 (8)	0.06573 (5)	0.0843 (2)	
N1	0.84486 (17)	0.86756 (18)	0.31082 (9)	0.0404 (3)	
N2	0.69901 (19)	1.0456 (2)	0.36035 (10)	0.0527 (4)	
N3	0.57837 (19)	0.9079 (2)	0.31201 (11)	0.0552 (4)	
N4	0.4093 (2)	0.4452 (2)	0.11718 (11)	0.0566 (4)	
C1	0.8562 (2)	1.0182 (2)	0.36077 (11)	0.0422 (4)	
C2	1.0238 (2)	1.1181 (2)	0.40777 (11)	0.0424 (4)	
C3	1.0384 (3)	1.2657 (2)	0.46264 (12)	0.0518 (5)	
H3	0.9390	1.3053	0.4684	0.062*	
C4	1.2006 (3)	1.3523 (2)	0.50822 (13)	0.0567 (5)	
H4	1.2103	1.4503	0.5452	0.068*	
C5	1.3493 (3)	1.2952 (3)	0.49973 (13)	0.0572 (5)	
H5	1.4581	1.3545	0.5313	0.069*	
C6	1.3375 (2)	1.1514 (3)	0.44506 (13)	0.0542 (5)	
H6	1.4388	1.1150	0.4390	0.065*	
C7	1.1733 (2)	1.0589 (2)	0.39819 (11)	0.0444 (4)	
C8	1.1543 (2)	0.9065 (2)	0.34138 (12)	0.0466 (4)	
H8	1.2558	0.8710	0.3341	0.056*	
C9	0.9971 (2)	0.8117 (2)	0.29783 (11)	0.0423 (4)	
C10	0.9794 (2)	0.6564 (2)	0.23864 (11)	0.0428 (4)	
C11	1.0353 (2)	0.5235 (3)	0.27137 (13)	0.0519 (5)	
H11	1.0870	0.5347	0.3299	0.062*	
C12	1.0157 (3)	0.3744 (3)	0.21852 (14)	0.0573 (5)	
H12	1.0532	0.2853	0.2412	0.069*	
C13	0.9398 (3)	0.3592 (3)	0.13168 (13)	0.0541 (5)	
C14	0.8902 (2)	0.4919 (3)	0.09643 (12)	0.0527 (5)	
H14	0.8433	0.4820	0.0371	0.063*	
C15	0.9105 (2)	0.6405 (2)	0.14989 (12)	0.0475 (4)	
H15	0.8776	0.7311	0.1262	0.057*	
C16	0.6634 (2)	0.8009 (2)	0.28300 (11)	0.0453 (4)	
C17	0.5732 (2)	0.6376 (2)	0.23238 (12)	0.0471 (4)	
C18	0.4858 (2)	0.6136 (2)	0.14858 (12)	0.0498 (5)	
C19	0.4738 (3)	0.7494 (3)	0.09026 (15)	0.0764 (7)	
H19A	0.3563	0.7645	0.0884	0.115*	
H19B	0.4969	0.7176	0.0312	0.115*	
H19C	0.5605	0.8544	0.1132	0.115*	
C20	0.4383 (2)	0.3425 (3)	0.17513 (14)	0.0558 (5)	
C21	0.3769 (4)	0.1526 (3)	0.16023 (18)	0.0799 (7)	
H21A	0.2505	0.1137	0.1641	0.120*	
H21B	0.4382	0.1043	0.2048	0.120*	
H21C	0.4023	0.1173	0.1023	0.120*	

### Atomic displacement parameters (Å^2^)

**Table:**

	*U*^11^	*U*^22^	*U*^33^	*U*^12^	*U*^13^	*U*^23^
S1	0.0593 (3)	0.0633 (4)	0.0552 (3)	0.0153 (3)	0.0034 (2)	0.0122 (2)
Cl1	0.0961 (5)	0.0658 (4)	0.0907 (5)	0.0316 (3)	−0.0026 (3)	−0.0189 (3)
N1	0.0364 (8)	0.0490 (9)	0.0405 (8)	0.0185 (7)	0.0070 (6)	0.0052 (6)
N2	0.0451 (9)	0.0614 (10)	0.0577 (10)	0.0276 (8)	0.0029 (7)	−0.0021 (8)
N3	0.0402 (9)	0.0680 (11)	0.0614 (10)	0.0249 (8)	0.0008 (7)	−0.0027 (8)
N4	0.0550 (10)	0.0589 (11)	0.0559 (10)	0.0203 (9)	−0.0029 (8)	−0.0027 (8)
C1	0.0411 (10)	0.0502 (10)	0.0406 (9)	0.0200 (8)	0.0084 (7)	0.0062 (8)
C2	0.0439 (10)	0.0467 (10)	0.0384 (9)	0.0135 (8)	0.0085 (7)	0.0095 (8)
C3	0.0581 (12)	0.0523 (11)	0.0503 (11)	0.0232 (10)	0.0079 (9)	0.0074 (9)
C4	0.0691 (13)	0.0485 (11)	0.0486 (11)	0.0115 (10)	0.0044 (9)	0.0009 (9)
C5	0.0509 (12)	0.0561 (13)	0.0566 (12)	0.0024 (10)	0.0006 (9)	0.0088 (10)
C6	0.0384 (10)	0.0640 (13)	0.0588 (12)	0.0106 (9)	0.0084 (8)	0.0072 (10)
C7	0.0399 (10)	0.0520 (11)	0.0435 (10)	0.0135 (8)	0.0111 (7)	0.0098 (8)
C8	0.0342 (9)	0.0601 (12)	0.0501 (10)	0.0192 (9)	0.0110 (8)	0.0049 (9)
C9	0.0364 (9)	0.0548 (11)	0.0422 (9)	0.0209 (8)	0.0116 (7)	0.0083 (8)
C10	0.0362 (9)	0.0540 (11)	0.0435 (10)	0.0190 (8)	0.0112 (7)	0.0054 (8)
C11	0.0526 (11)	0.0644 (13)	0.0468 (11)	0.0290 (10)	0.0064 (8)	0.0078 (9)
C12	0.0618 (12)	0.0573 (12)	0.0628 (13)	0.0308 (11)	0.0119 (10)	0.0114 (10)
C13	0.0511 (11)	0.0549 (12)	0.0586 (12)	0.0183 (10)	0.0109 (9)	−0.0014 (9)
C14	0.0518 (11)	0.0665 (13)	0.0436 (10)	0.0232 (10)	0.0066 (8)	0.0011 (9)
C15	0.0457 (10)	0.0586 (12)	0.0454 (10)	0.0242 (9)	0.0099 (8)	0.0085 (9)
C16	0.0344 (9)	0.0608 (12)	0.0442 (10)	0.0189 (9)	0.0041 (7)	0.0053 (9)
C17	0.0354 (9)	0.0592 (12)	0.0495 (11)	0.0181 (9)	0.0048 (8)	0.0037 (9)
C18	0.0460 (10)	0.0538 (11)	0.0521 (11)	0.0215 (9)	−0.0021 (8)	−0.0002 (9)
C19	0.0994 (17)	0.0625 (14)	0.0660 (14)	0.0318 (13)	−0.0244 (12)	0.0001 (11)
C20	0.0501 (11)	0.0564 (12)	0.0623 (13)	0.0169 (10)	0.0088 (9)	0.0039 (10)
C21	0.0942 (18)	0.0585 (14)	0.0864 (17)	0.0212 (13)	0.0081 (13)	0.0040 (12)

### Geometric parameters (Å, °)

**Table:**

S1—C17	1.7150 (19)	C8—C9	1.351 (2)
S1—C20	1.722 (2)	C8—H8	0.9300
Cl1—C13	1.7387 (19)	C9—C10	1.475 (2)
N1—C1	1.382 (2)	C10—C11	1.382 (2)
N1—C16	1.392 (2)	C10—C15	1.389 (2)
N1—C9	1.413 (2)	C11—C12	1.381 (3)
N2—C1	1.310 (2)	C11—H11	0.9300
N2—N3	1.376 (2)	C12—C13	1.379 (3)
N3—C16	1.313 (2)	C12—H12	0.9300
N4—C20	1.299 (3)	C13—C14	1.371 (3)
N4—C18	1.378 (2)	C14—C15	1.381 (2)
C1—C2	1.441 (2)	C14—H14	0.9300
C2—C3	1.394 (2)	C15—H15	0.9300
C2—C7	1.399 (2)	C16—C17	1.460 (2)
C3—C4	1.374 (3)	C17—C18	1.365 (3)
C3—H3	0.9300	C18—C19	1.477 (3)
C4—C5	1.380 (3)	C19—H19A	0.9600
C4—H4	0.9300	C19—H19B	0.9600
C5—C6	1.372 (2)	C19—H19C	0.9600
C5—H5	0.9300	C20—C21	1.490 (3)
C6—C7	1.405 (3)	C21—H21A	0.9600
C6—H6	0.9300	C21—H21B	0.9600
C7—C8	1.435 (2)	C21—H21C	0.9600
			
C17—S1—C20	89.71 (9)	C12—C11—H11	119.5
C1—N1—C16	104.18 (13)	C10—C11—H11	119.5
C1—N1—C9	122.02 (14)	C13—C12—C11	119.11 (18)
C16—N1—C9	133.80 (15)	C13—C12—H12	120.4
C1—N2—N3	106.96 (14)	C11—C12—H12	120.4
C16—N3—N2	109.14 (14)	C14—C13—C12	121.03 (18)
C20—N4—C18	111.44 (17)	C14—C13—Cl1	119.56 (16)
N2—C1—N1	110.74 (15)	C12—C13—Cl1	119.41 (16)
N2—C1—C2	128.52 (16)	C13—C14—C15	119.30 (18)
N1—C1—C2	120.68 (14)	C13—C14—H14	120.3
C3—C2—C7	120.43 (17)	C15—C14—H14	120.3
C3—C2—C1	122.28 (16)	C14—C15—C10	120.84 (18)
C7—C2—C1	117.26 (16)	C14—C15—H15	119.6
C4—C3—C2	119.62 (18)	C10—C15—H15	119.6
C4—C3—H3	120.2	N3—C16—N1	108.91 (16)
C2—C3—H3	120.2	N3—C16—C17	123.14 (15)
C3—C4—C5	120.64 (19)	N1—C16—C17	127.92 (15)
C3—C4—H4	119.7	C18—C17—C16	126.79 (17)
C5—C4—H4	119.7	C18—C17—S1	109.80 (14)
C6—C5—C4	120.41 (19)	C16—C17—S1	123.38 (14)
C6—C5—H5	119.8	C17—C18—N4	114.72 (17)
C4—C5—H5	119.8	C17—C18—C19	125.94 (18)
C5—C6—C7	120.40 (18)	N4—C18—C19	119.29 (17)
C5—C6—H6	119.8	C18—C19—H19A	109.5
C7—C6—H6	119.8	C18—C19—H19B	109.5
C2—C7—C6	118.48 (17)	H19A—C19—H19B	109.5
C2—C7—C8	119.24 (16)	C18—C19—H19C	109.5
C6—C7—C8	122.28 (16)	H19A—C19—H19C	109.5
C9—C8—C7	123.62 (16)	H19B—C19—H19C	109.5
C9—C8—H8	118.2	N4—C20—C21	124.4 (2)
C7—C8—H8	118.2	N4—C20—S1	114.31 (16)
C8—C9—N1	116.99 (16)	C21—C20—S1	121.26 (17)
C8—C9—C10	123.21 (15)	C20—C21—H21A	109.5
N1—C9—C10	119.80 (14)	C20—C21—H21B	109.5
C11—C10—C15	118.60 (17)	H21A—C21—H21B	109.5
C11—C10—C9	119.53 (16)	C20—C21—H21C	109.5
C15—C10—C9	121.87 (16)	H21A—C21—H21C	109.5
C12—C11—C10	121.00 (18)	H21B—C21—H21C	109.5
			
C1—N2—N3—C16	−0.8 (2)	N1—C9—C10—C15	57.7 (2)
N3—N2—C1—N1	2.2 (2)	C15—C10—C11—C12	−3.1 (3)
N3—N2—C1—C2	−174.89 (17)	C9—C10—C11—C12	178.05 (16)
C16—N1—C1—N2	−2.68 (19)	C10—C11—C12—C13	0.3 (3)
C9—N1—C1—N2	177.60 (14)	C11—C12—C13—C14	2.5 (3)
C16—N1—C1—C2	174.71 (15)	C11—C12—C13—Cl1	−178.02 (15)
C9—N1—C1—C2	−5.0 (2)	C12—C13—C14—C15	−2.5 (3)
N2—C1—C2—C3	0.4 (3)	Cl1—C13—C14—C15	178.05 (13)
N1—C1—C2—C3	−176.44 (16)	C13—C14—C15—C10	−0.4 (3)
N2—C1—C2—C7	178.86 (17)	C11—C10—C15—C14	3.1 (3)
N1—C1—C2—C7	2.0 (2)	C9—C10—C15—C14	−178.05 (15)
C7—C2—C3—C4	−0.7 (3)	N2—N3—C16—N1	−0.8 (2)
C1—C2—C3—C4	177.71 (16)	N2—N3—C16—C17	177.39 (16)
C2—C3—C4—C5	0.4 (3)	C1—N1—C16—N3	2.09 (18)
C3—C4—C5—C6	0.4 (3)	C9—N1—C16—N3	−178.24 (17)
C4—C5—C6—C7	−1.1 (3)	C1—N1—C16—C17	−176.03 (17)
C3—C2—C7—C6	0.1 (3)	C9—N1—C16—C17	3.6 (3)
C1—C2—C7—C6	−178.40 (15)	N3—C16—C17—C18	66.9 (3)
C3—C2—C7—C8	179.91 (16)	N1—C16—C17—C18	−115.2 (2)
C1—C2—C7—C8	1.4 (2)	N3—C16—C17—S1	−111.10 (18)
C5—C6—C7—C2	0.8 (3)	N1—C16—C17—S1	66.8 (2)
C5—C6—C7—C8	−179.03 (17)	C20—S1—C17—C18	1.01 (14)
C2—C7—C8—C9	−2.1 (3)	C20—S1—C17—C16	179.31 (15)
C6—C7—C8—C9	177.69 (17)	C16—C17—C18—N4	−178.77 (15)
C7—C8—C9—N1	−0.7 (3)	S1—C17—C18—N4	−0.5 (2)
C7—C8—C9—C10	179.18 (16)	C16—C17—C18—C19	3.7 (3)
C1—N1—C9—C8	4.3 (2)	S1—C17—C18—C19	−178.11 (17)
C16—N1—C9—C8	−175.35 (17)	C20—N4—C18—C17	−0.5 (2)
C1—N1—C9—C10	−175.59 (15)	C20—N4—C18—C19	177.28 (18)
C16—N1—C9—C10	4.8 (3)	C18—N4—C20—C21	−178.43 (18)
C8—C9—C10—C11	56.7 (2)	C18—N4—C20—S1	1.3 (2)
N1—C9—C10—C11	−123.46 (18)	C17—S1—C20—N4	−1.35 (15)
C8—C9—C10—C15	−122.2 (2)	C17—S1—C20—C21	178.36 (17)

### Hydrogen-bond geometry (Å, °)

**Table:**

*D*—H···*A*	*D*—H	H···*A*	*D*···*A*	*D*—H···*A*
C6—H6···N2^i^	0.93	2.62	3.495 (2)	158
C8—H8···N3^i^	0.93	2.51	3.383 (2)	156

Symmetry codes: (i) *x*+1, *y*, *z*.
